# Twin Screw Granulation: Effects of Properties of Primary Powders

**DOI:** 10.3390/pharmaceutics10020068

**Published:** 2018-06-02

**Authors:** Sushma V. Lute, Ranjit M. Dhenge, Agba D. Salman

**Affiliations:** Department of Chemical and Biological Engineering, University of Sheffield, Mappin Street, Sheffield S1 3JD, UK; sushmalute@gmail.com (S.V.L.); ranjitdhenge@gmail.com (R.M.D.)

**Keywords:** twin screw wet granulation, continuous, excipient, granule, lactose, mannitol, tableting

## Abstract

Lactose and mannitol are some of the most commonly used powders in the pharmaceutical industry. The limited research published so far highlights the effects of process and formulation parameters on the properties of the granules and the tablets produced using these two types of powders separately. However, the comparison of the performance of these two types of powders during twin screw wet granulation has received no attention. The present research is focused on understanding the granulation mechanism of different grades of two pharmaceutical powders with varying properties (i.e., primary particle size, structure, and compressibility). Three grades each of lactose and mannitol were granulated at varying liquid to solid ratios (L/S) and screw speed. It was noticed that primary powder morphology plays an important role in determining the granule size and structure, and tablet tensile strength. It was indicated that the processed powders such as spray-dried and granulated lactose and mannitol can be used in formulation for wet granulation where flowability of active pharmaceutical ingredient (API) is poor.

## 1. Introduction

In the pharmaceutical industry, granulation of powders to form structured products (i.e., granules) is one of the most important operations in solid oral dosage form manufacturing to improve flow, compaction or homogeneity of powders. It can be carried out using either dry or wet approaches. In wet granulation, liquid binder is added onto a bed of primary powder particles that is being agitated by an impeller (in a high-shear granulator (HSG), screws (in twin screw granulator (TSG), or air (in a fluidized bed granulator (FBG)) to form wet granules.

Amongst various excipient (filler/diluent) powders available for the wet/dry granulation and tableting in the pharmaceutical industry, lactose and mannitol are some of the most common ones. Both lactose and mannitol are commercially available in different grades (e.g., sieved, milled, spray-dried, granulated, etc.) presenting different particle characteristics (i.e., size, size distribution, degrees of fines, shape, surface, flowability, and compressibility) [1,2].

Lactose occurs in α and β forms possessing different melting points, solubility, and hardness [3]. It is known that different grades of lactose have different granulation and compression properties [3,4]. For example, sieved α-lactose monohydrate is used in direct compression, whereas milled α-lactose monohydrate is wet granulated prior to tableting due to its poor compressibility under direct compaction [2]. Spray-dried lactose and anhydrous lactose are recommended for dry granulation and direct compaction owing to their excellent flowability and compressibility [1].

Mannitol is used as a filler/diluent in tablet making with both wet/dry granulation and direct compaction methods in the pharmaceutical industry [5]. Mannitol, due to its non-hygroscopic nature, sweet taste, and negative heat of the solution giving a cooling sensation, is a preferred excipient for formulating the moisture-sensitive drugs [6] and producing chewable tablets or lozenges [7]. Mannitol is known to exist in different polymorphic forms including the α, β, and δ having different compressibility [8]. Like lactose, mannitol is also manufactured in different grades using different approaches for specific application, hence, the different size, shape, and structure. For example, crystalline mannitol is a brittle powder with needle-shaped particles used in wet granulation [9]. Spray-dried mannitol is a crystalline, highly brittle granular powder (high compressibility) with spherical particles used in dry granulation (roller compaction) and direct compaction (tableting) [10]. Granulated mannitol is a crystalline, brittle mannitol powder with high compressibility making it suitable for direct compaction [8].

The particle characteristics of lactose and mannitol powders can play important roles in granulation. For instance, primary powder particle size plays a critical role in determining the size of nuclei during the nucleation step in wet granulation [11], which further influences the final granules’ attributes [12,13]. Thus, the effect of particle size and other characteristics of primary powders need to be considered during product development using a wet granulation platform.

In batch high-shear granulation, the effect of primary powder morphology on granulation behavior and properties of granules and tablets have been studied previously [3,4,5,6]. Kristensen, et al. [14] investigated the influence of primary particle size of dicalcium phosphate powder on the granules’ growth. They found that the granule growth was inversely proportional to the particle size of dicalcium phosphate powder. Badawy, et al. [15] also reported a similar tendency for their powder of interest (code name: DPC 963). They also noticed that the granule size, porosity, and compressibility were inversely proportional to the powder particle size. The smaller powder particles showed reduced tendency for densification than the larger ones. Mackaplow, et al. [16] studied the effect of varying primary particles size of lactose monohydrate powder on granule growth and end point determination during high-shear wet granulation. Unlike Kristensen, et al. [14] and Badawy, et al. [15], Mackaplow, et al. [16] and Badawy and Hussain [17] found that increasing primary particle size of lactose powder results in larger, less porous wet granules. This was attributed to the decrease in both the capillary and viscous interparticle forces with increasing primary particle size making granules more deformable. This difference in the observations in two studies may be attributed to the type of powder used. Keleb, et al. [3] also studied the effect of primary particle size (different grades of milled lactose: Pharmatose 450M, Pharmatose 200M, Pharmatose 100M, and Pharmatose 90M); morphology (anhydrous β-lactose (Pharmatose DCL 21); spray-dried lactose (Pharmatose DCL 11); and milled lactose (Pharmatose 90M)) of lactose powder on properties of tablets (friability, tensile strength, and disintegration time) produced from compression of granules after batch high-shear wet granulation and single-step granulation/tableting (extrusion). In cases of batch high-shear wet granulation, they noticed that the tablet friability and disintegration time decreased and tensile strength increased first with increasing primary particle size (from 450M to 200M), and then the trend was reversed (in cases of 100M and 90M). The morphology of lactose powders also influenced the disintegration time and tensile strength of tablets. The tensile strength of tablets produced from spray-dried and anhydrous lactose powders was similar (stronger than 90 M); however, they differed in terms of disintegration time where spray dried lactose tablets disintegrated slower compared to other powders. The reasons for such trends needed further justifications.

Huang, et al. [1] compared the suitability of four commercial grades of lactose (spray-dried/sieved/milled monohydrate and anhydrous lactose) for a low-dose oral formulation of pentyloxyl paliperidone derivative with drug loading at 1.5% (*w*/*w*) and lower using a batch high-shear granulator. The effects on granule size, flowability, and other product attributes were investigated. It was noticed that granule size increases with an increase in the particle size primary powder. It was also found that spray-dried lactose powder produce granules with better flowability, narrow granule, size distribution, and tablets with good hardness and low friability. It can also reduce the degree segregation/agglomeration of granules throughout the manufacturing process.

Compared to batch high-shear wet granulation, the effect of primary powder (i.e., lactose and mannitol) morphology on granulation behavior and properties of granules and tablets has received limited attention in continuous twin screw wet granulation [18,19]. Moreover, the comparison of the performance of different grades of lactose vs. mannitol powders in twin screw wet granulation has not been studied previously. El Hagrasy, et al. [18] granulated three different grades of lactose powder (Pharmatose 200M, Supertab 30GR, and Impalpable) as a major ingredient mixed with three minor ingredients, namely microcrystalline cellulose, hydroxypropylmethyl cellulose, and croscarmellose sodium at different liquid to solid ratios (L/S) in TSG. They concluded that the changes in the lactose grades in the formulation displayed comparable growth behavior at different L/S, while impact of particle size/grade type on granule porosity was inconclusive. 

It is only recently that Vanhoorne, et al. [19] investigated the impact of using different polymorphs of mannitol powder (i.e., δ-mannitol (Parteck Delta M), β-mannitol (C*PharmMannidex 16700), and α-mannitol (Pearlitol 200)) on the granules and tablet attributes in twin screw wet granulation. They observed that δ-mannitol changes into β-mannitol during wet granulation leading to a unique granule morphology with a higher specific surface area and better plastic deformability compared to α- and β-mannitol as starting material. 

The objective of this study was to compare the performance of different grades of lactose and mannitol powders during twin screw wet granulation. Different types of lactose and mannitol powders having different powder morphology were granulated separately at different L/S and screw speeds and their impact on the properties of the granules (size, shape, and structure) and tablet (tensile strength) was investigated.

## 2. Materials and Methods

### 2.1. Materials

#### 2.1.1. Powder

Different grades of lactose and mannitol powders were used in this study for comparing the effects of their physical properties on the granulation behavior and granule properties.

Three types of lactose powders were used in the study: α-lactose monohydrate (Pharmatose 200M), spray-dried lactose (SuperTab11SD), and anhydrous lactose (SuperTab21AN). All powders were supplied by DMV-Fonterra Excipient GmbH and co., Goch, Germany. α-lactose monohydrate powder is manufactured by slow crystallization of a supersaturated lactose solution below 93.5 °C accompanied by roller drying resulting in single crystals of α-lactose monohydrate. The crystals are further milled to produce Pharmatose 200M grade powder (amorphous lactose ~2.6% [20]). Anhydrous lactose is also made from crystallization of a supersaturated solution of lactose, but it is rapidly dried at high temperature (above 93.5 °C) by roller drying. The particles are then milled and sieved to produce SuperTab21AN grade powder. SuperTab21AN particles consist of clusters of micro-crystals of predominantly anhydrous β-lactose together with anhydrous α-lactose [21]. SuperTab21AN contains negligible amounts of amorphous lactose (~0.2%) [20]. Spray-dried lactose consists of particles of α-lactose monohydrate in a matrix of amorphous lactose (~10 to 15%) [20,22]. Finely-milled α-lactose monohydrate is suspended in water and spray dried to make spherical agglomerates to produce SuperTab11SD.

The three types of mannitol powders, used in respective comparison with the three lactose powders, were milled crystalline mannitol (Pearlitol 50C) (β-mannitol [23]), spray-dried mannitol (Pearlitol 200SD) (known to contain both α- and β-mannitol [24] and is crystalline [25]), and granulated mannitol (Pearlitol 300DC) (β-mannitol [8,23]). All mannitol powders were supplied by Roquette (Lestrem cedex, France). Milled crystalline mannitol powder is manufactured by slow crystallization of a supersaturated solution to produce crystals of mannitol which are then crushed to produce Pearlitol 50C. Spray-dried mannitol, as the name suggests, is manufactured by spray drying a suspension of mannitol powder in water to produce spherical agglomerates. The granulated mannitol is produced by pouring a hot slurry of mannitol powder on the cold rotating drum and then crushing into the desired size.

#### 2.1.2. Granulator

A co–rotating twin screw granulator (TSG) (Euro lab 16 TSG (L/D-25/1), Prism, Thermo Scientific (Thermo Electron GmbH), Karlsruhe, Germany) was used for the granulation experiments. A gravimetric, loss-in-weight twin screw powder feeder (K-PH-CL-24-KT20, K-Tron Soder, Niederlenz, Switzerland) having a pair of co-rotating screws was used to feed powder into the granulator. A peristaltic pump (101U, Watson Marlow, Cornwall, UK) was used to inject granulation liquid (distilled water) into the granulator.

### 2.2. Method

#### 2.2.1. Morphology of Powders

Powder particle size was measured using Camsizer-XT (X-jet module) (Retsch Technology, Haan, Germany). The powder shape and surface were examined using scanning electron microscopy (SEM) (Jeol, Peabody, MA, USA). The lactose and mannitol particles were nonconductive, hence, were coated (for 40 s) with a thin layer (~25 nm) of gold using a coating machine (Leica EM ACE200, Leica Microsystems, Milton Keynes, UK).

#### 2.2.2. Compressibility Factor for Powders

The compressibility of factor *K* for all six powders was determined from the relationship between the uniaxial stress σ and the resulting powder bed apparent density *ρ*, as shown in Equation (1) [4]. It is known that the lower the compressibility factor, the more compressible the powder is [4]. This was measured by compressing 405 mg of powders in a die (diameter: 12 mm) using an Instron testing machine (Instron 3367, High Wycombe, Buckinghamshire, UK) under a range of compression forces (1000 to 5000 N) at test speed of 1 mm/min. and determining the apparent density of the tablet (from mass and volume measured out of die (i.e., post tablet ejection, after 24 h)). The compressibility factor *K* was determined from the slope of a logarithmic plot of the apparent density as a function of stress.

(1) $$\frac{\sigma_{1}}{\sigma_{2}}={\left(\frac{\rho_{1}}{\rho_{2}}\right)}^{K}$$

where,

*σ*_1_ = major principal stress
*σ*_2_ = minor principal stress
*ρ*_1_ = powder bulk density at *σ*_1_
*ρ*_2_ = powder bulk density at *σ*_2_

#### 2.2.3. Preparation of Granules

**Figure 1:**
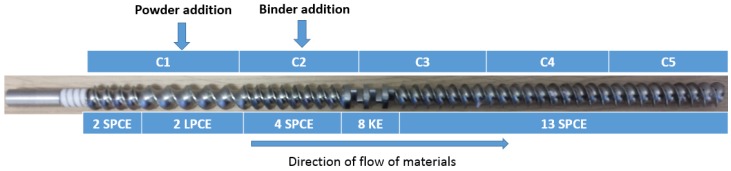
Screw configuration used in the experiments (SPCE, short pitch conveying element; LPCE, long pitch conveying element; KE, kneading element; C1 to C5, compartments/ports along the length of the barrel).

Lactose and mannitol powders were granulated separately using distilled water in the TSG. The granules (60 g in each experiment) were collected after 1 min, when the system reached the equilibrium (based on the stabilization time (~30 s at powder feed rate of 2 kg/h), which was determined by monitoring feed factor variability over time) for a gravimetric, loss-in-weight twin screw powder feeder. The granulation was carried out using the full length of the granulator keeping the screw configuration unchanged (Figure 1).

The experimental design is shown in Table 1. In total, 288 experiments (i.e., 16 combinations × 3 repetitions of each combination × 6 powders) were carried out. Each powder was granulated at 4 different L/S and 4 different screw speeds in order to compare its performance under varying process variables, and its effects on the properties of granules (i.e., size, shape, and structure) and tablets (tensile strength).

#### 2.2.4. Size Analysis of Granules

The granules were air dried at room temperature for 48 h. The median size (d_50_) of the granules was measured using the particle size analyzer: Camsizer (free-fall module) (Retsch Technology, Haan, Germany). Three repetitions were performed for the size analysis.

#### 2.2.5. Structural Analysis of Granules

The X-ray tomography (XRT) (µCT 35, SCANCO Medical AG, Brüttisellen, Switzerland) of granules was carried out to gain more information on the change in the internal structure of the granules. The granules collected from various processing conditions had varying shapes. For this reason, only the middle area of the granules was scanned to determine the porosity. The stack of 240 images (slices) was threshold to differentiate the void and solid particle using ImageJ Software (National Institutes of Health, Bethesda, MD, USA) [26]. The black area in the X-ray images indicates the air (pores), and the white area indicates the granule or powder particle. The porosity of a granule was determined by dividing the area of air by the total area of the image.

#### 2.2.6. Tableting

The granules produced at two extremes of L/S (i.e., 0.048 and 0.113) at 4 screw speeds were sieved into different size classes (212–600 µm, 600–1000 µm, 1000–1400 µm, and 212–1400 µm) and compressed in a 12 mm die at 10 kN compression force (test speed, 1 mm/min) to produce tablets of about 405 mg using an Instron testing machine. This was done to study the impact of granule size range on the tablet tensile strength [3]. The granules were not pre-lubricated internally or externally; however, the punch and die of the compression machine were coated with a thin layer of magnesium stearate to minimize sticking and picking.

#### 2.2.7. Analysis of Tablets

The tablets so produced were analyzed for their dimensions (i.e., thickness and diameter) and tensile strength. The thickness and diameter were measured using a digital caliper. The tensile strength of tablets was measured by diametric compression method using Zwick/Roell Z 0.5 (Zwick/Roell, Ulm, Germany) instead of an Instron testing machine. This was done because Zwick/Roell provides more suitable force range compared to the Instron testing machine. The tablets were compressed diametrically (test speed, 1 mm/min) until they fractured. The force-displacement data was recorded. Ten tablets were used for each experimental condition to produce reproducible data. The strength of tablets (*σ)* was determined by inputting maximum force (*F*), tablet diameter (*D*), and thickness (*T*) in Equation (2) [27,28].

(2) $$\sigma =2\frac{F}{\pi TD}$$

## 3. Results

### 3.1. Morphology of Powder

The morphology of powder (i.e., size and shape) is discussed in this section. The primary particle size of six different powders is shown in Table 2. It can be seen that SuperTab21AN and Pearlitol 300DC had the largest particle size (d_50_) amongst lactose and mannitol powders, respectively.

**Figure 2:**
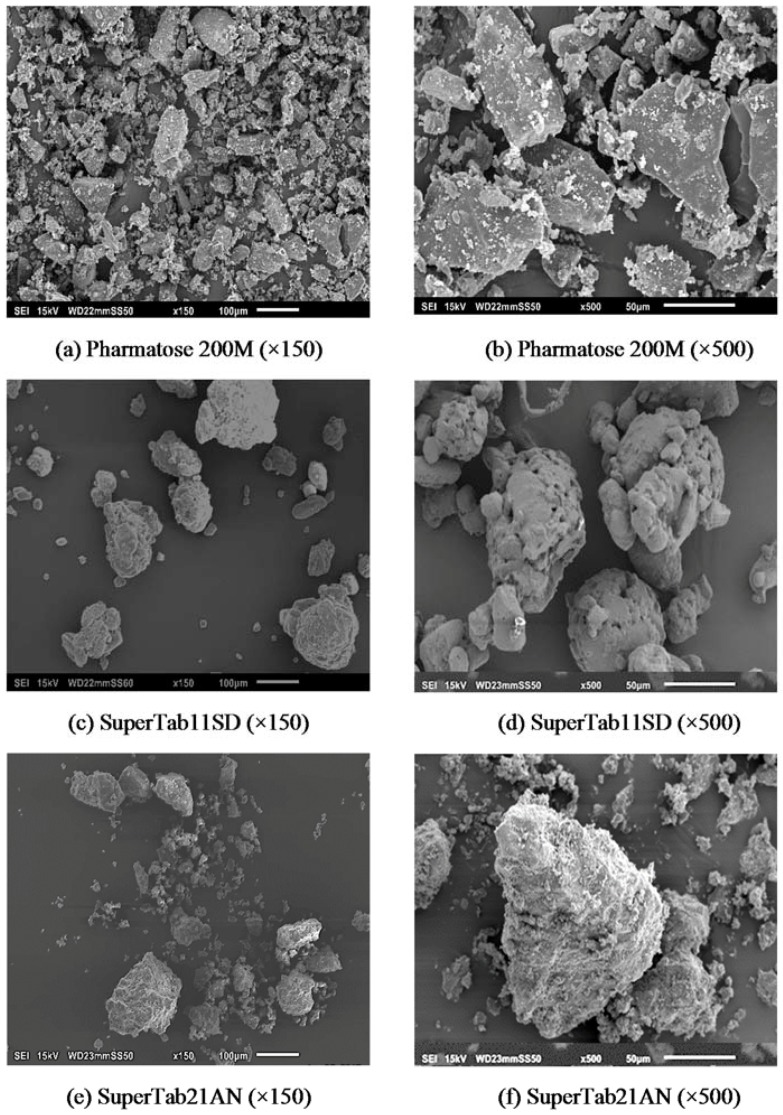
Scanning Electron Microscope (SEM) images of lactose powder.

**Figure 3:**
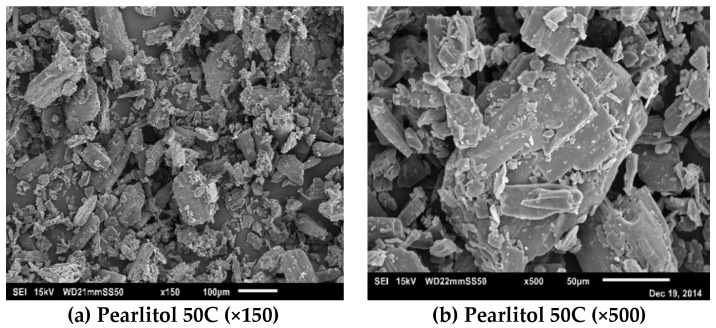

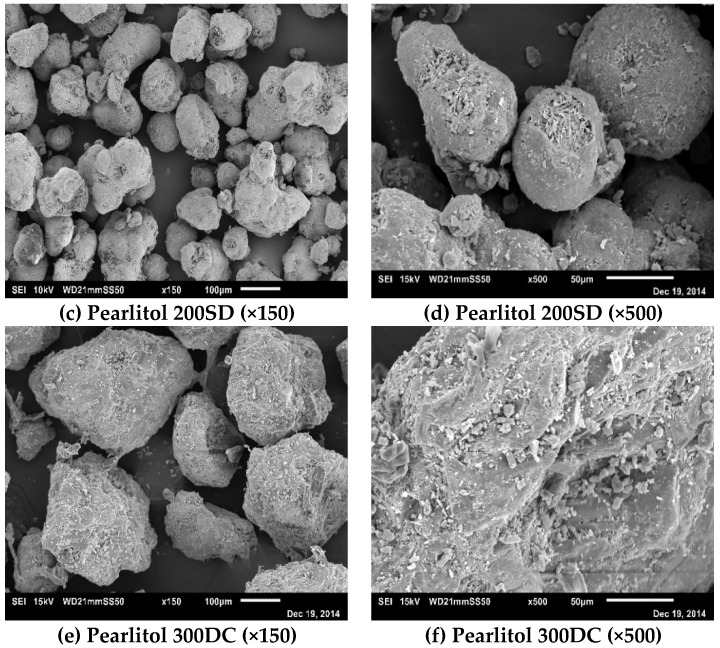
SEM images of mannitol powders.

The scanning electron microscopy (SEM) images for lactose and mannitol powders are shown in Figure 2 and Figure 3, respectively. The SEM images for lactose and mannitol powders clearly show the difference in their size and shape. Pharmatose 200M is a crystalline milled lactose with tomahawk like shape (Figure 2a,b). SuperTab11SD is spray-dried lactose with porous and relatively spherical particles, and its structure is made from α-lactose monohydrate (Figure 2c,d). SuperTab21AN is crystalline anhydrous lactose, which is an aggregation of lactose microcrystals [29] (Figure 2e,f). Pearlitol 50C powder has elongated particles (Figure 3a,b) with smaller particles sticking on the larger ones. Pearlitol 200SD has more spherical shaped primary particles (Figure 3c,d) having porous shells with elongated thread-like smaller particles embedded in them [30]. Pearlitol 300DC is a granulated mannitol powder with compact and rounded particles (Figure 3e,f).

### 3.2. Compressibility Factor for Powders

**Figure 4:**
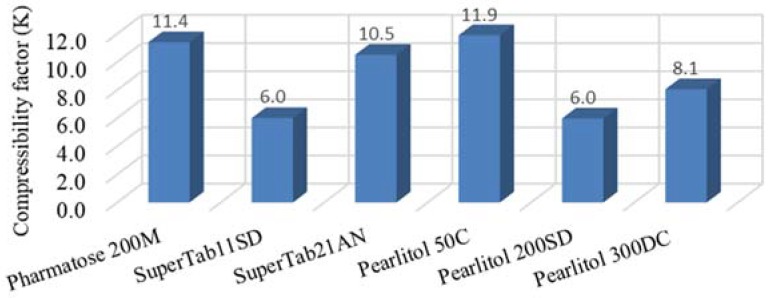
Compressibility factor for different powders.

Figure 4 shows compressibility factor (*K*) for different powders. The compressibility factor results for lactose and mannitol powders were anomalous. Based on the particle size, the expected trend for *K* value was Pharmatose 200M < SuperTab11SD < SuperTab21AN for lactose and Pearlitol 50C < Pearlitol 200SD < Pearlitol 300DC for mannitol; however, it was not the case. The *K* value was higher for crystalline Pharmatose 200M and Pearlitol 50C having smaller particle size, while it was lower for spray-dried large sized (meaning higher compressibility) SuperTab11SD and Pearlitol 200SD, respectively. It was also expected that the pre-processed SuperTab21AN and Pearlitol 300DC (which are suitable for direct compression in tableting) to have lower *K* than SuperTab11SD and Pearlitol 200SD, respectively, but it was not the case. This can be attributed to the structure of the spray-dried lactose and mannitol particles. Scanned electron microscopy images of SuperTab11SD particles (Figure 2c,d) show that they are brittle aggregates of several lactose microcrystals, which during compression or tableting fracture easily into several fine particles, which improve the compressibility (even better than SuperTab21AN). Similar is the case for spray-dried mannitol or Pearlitol 200SD, where the SEM images (Figure 3c,d) clearly show the presence of elongated, smaller particles embedded in the spherical shell. According to Mitra, et al. [31], during compression such Pearlitol 200SD particles may possibly break into smaller fragments and thereby improve compressibility. It can also be noticed from Figure 4 that Pearlitol 200SD has a lower *K* value (i.e., higher compressibility) compared to Pearlitol 300DC, which is a granular compact.

### 3.3. Size of Granules

#### 3.3.1. Lactose

**Figure 5:**
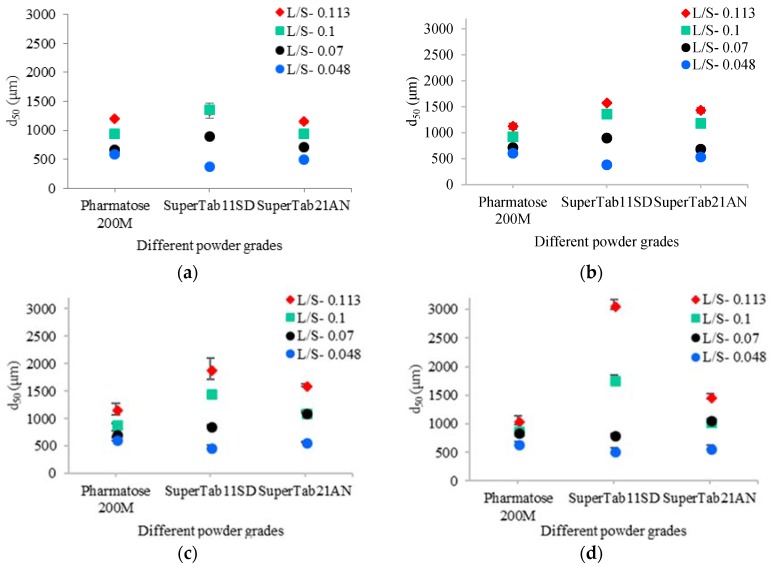
Median size of granules for different grades of lactose powder at varying L/S and screw speeds. (**a**) 200 rpm; (**b**) 450 rpm; (**c**) 700 rpm; (**d**) 950 rpm.

Figure 5a–d shows the median size of granules produced using different grades of lactose powder at varying L/S and screw speeds. For the ease of understanding the results, they are discussed in two parts viz. the effect of different powder properties on the granule size at varying L/S and the same at varying screw speed.

#### 3.3.2. Mannitol

**Figure 6:**
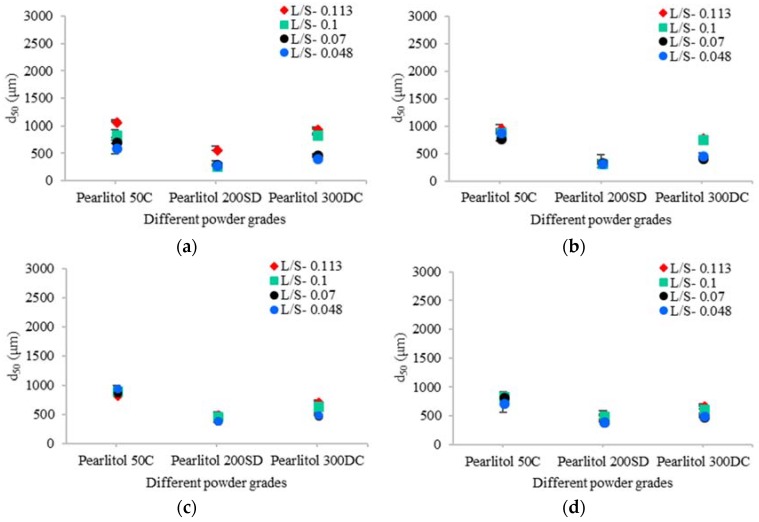
Median size of granules for different grades of mannitol powder at varying L/S and screw speeds. (**a**) 200 rpm; (**b**) 450 rpm; (**c**) 700 rpm; (**d**) 950 rpm.

In the case of mannitol (Figure 6a–d), increasing the primary particle size of powder at varying L/S and screw speeds had varying influences on the median granule size. Similar to lactose, for the ease of understanding the results they are discussed in two parts viz. the effect of increasing powder particle size on granule size at varying L/S and the same at varying screw speeds.

### 3.4. Structure of Granules

**Figure 7:**
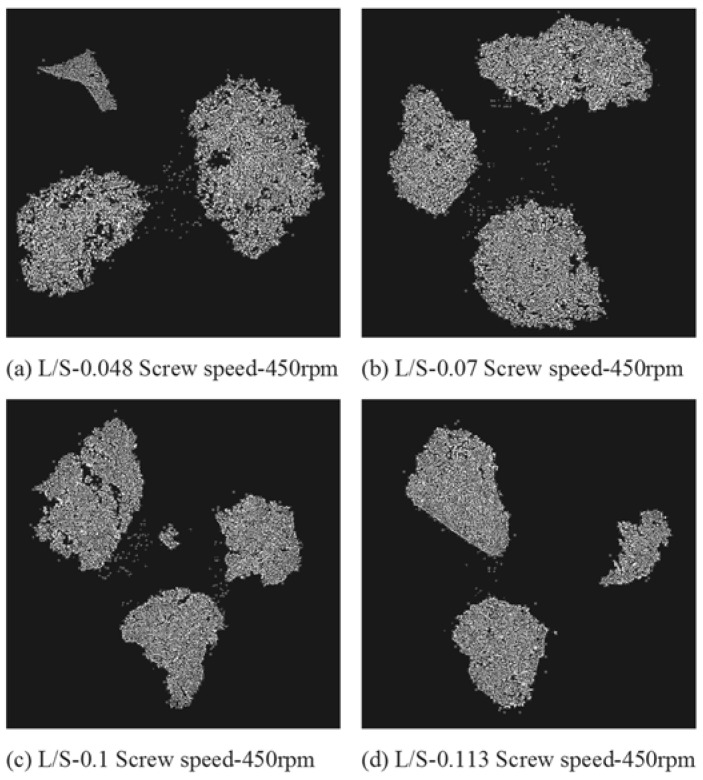
X-ray tomographic images of granules produced using Pharmatose 200M at varying L/S (screw speed, 450 rpm).

**Figure 8:**
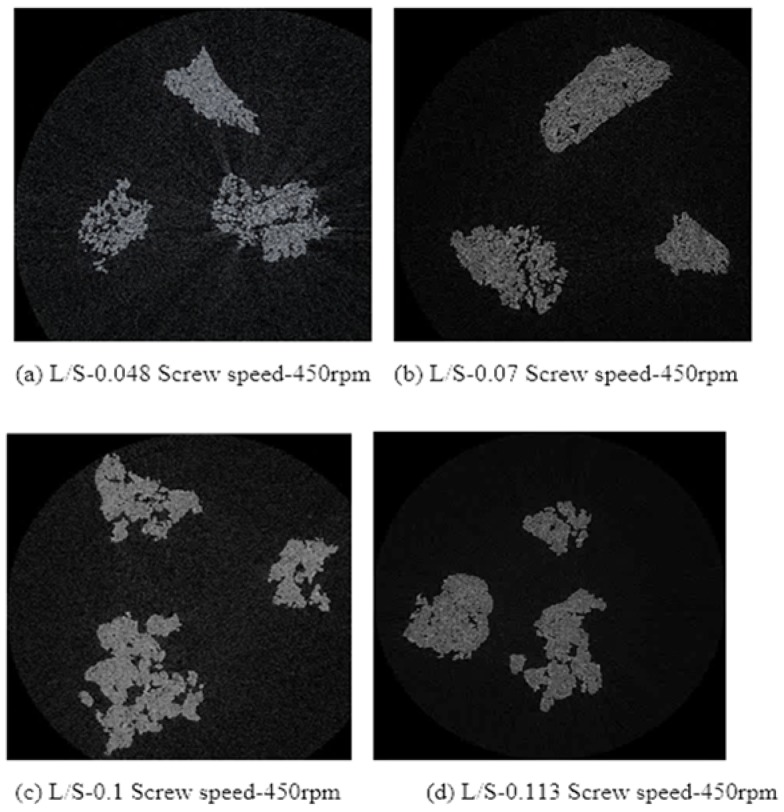
X-ray tomographic images of granules produced using SuperTab11SD at varying L/S (screw speed, 450 rpm).

**Figure 9:**
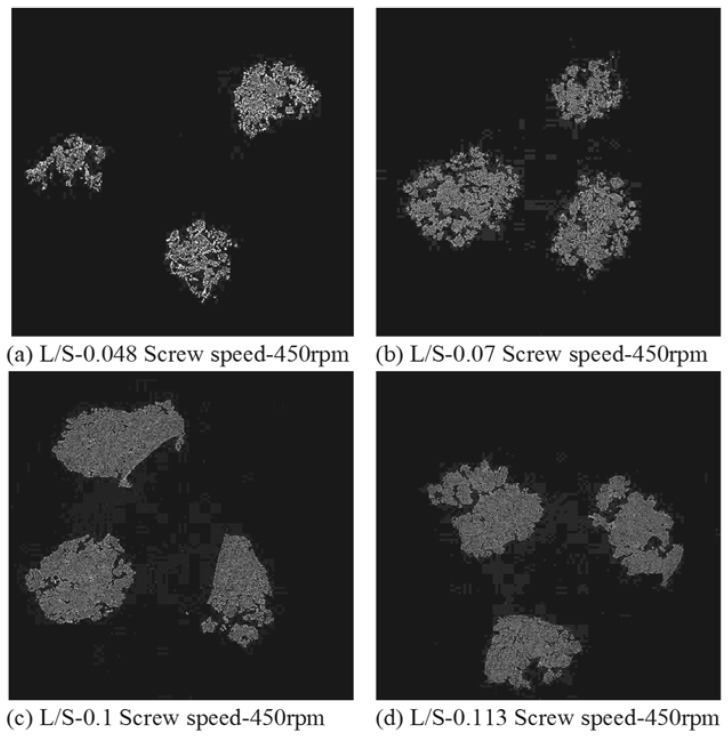
X-ray tomographic images of granules produced using SuperTab21AN at varying L/S (screw speed, 450 rpm).

**Figure 10:**
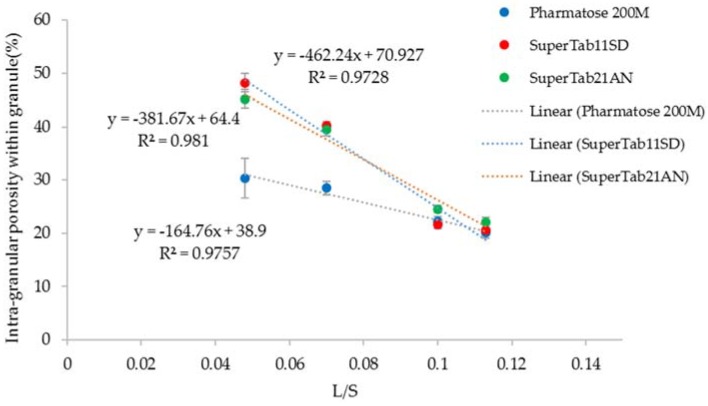
Porosity of granules for different grades of lactose powder at varying L/S (screw speed, 450 rpm).

Figure 7, Figure 8 and Figure 9 show the XRT images of granules produced using different lactose powders at varying L/S (constant screw speed, 450 rpm). The respective intra-granular porosity within the granules calculated using ImageJ software is presented in Figure 10.

**Figure 11:**
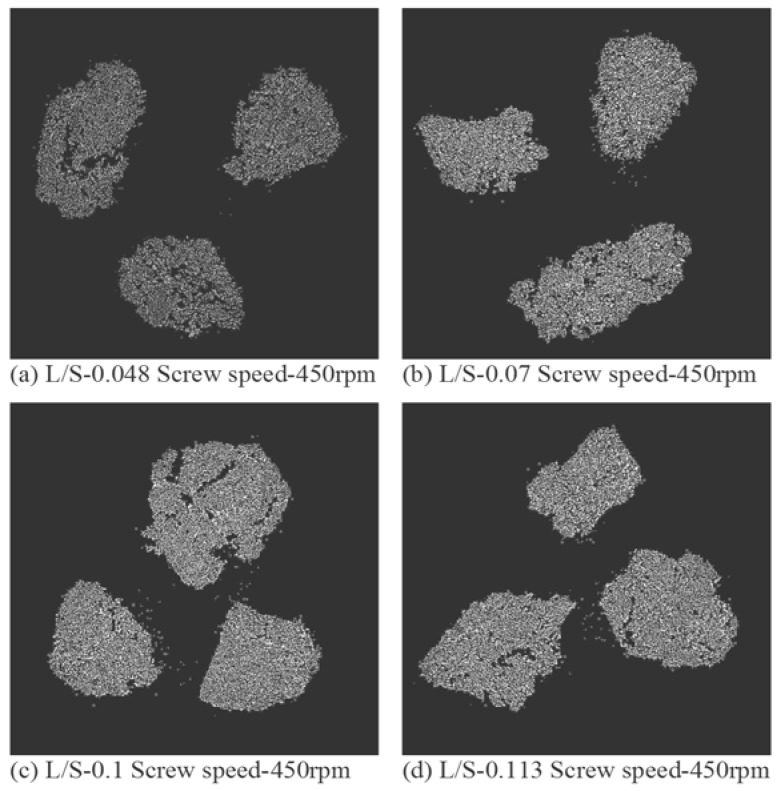
X-ray tomographic images of granules produced using Pearlitol 50C at varying L/S (screw speed, 450 rpm).

**Figure 12:**
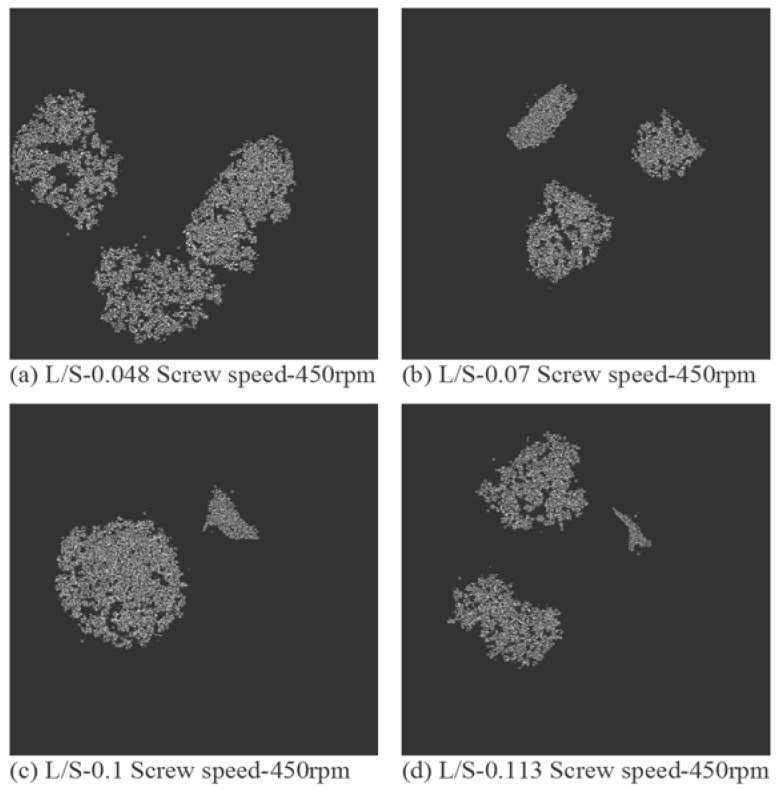
X-ray tomographic images of granules produced using Pearlitol 200SD at varying L/S (screw speed, 450 rpm).

**Figure 13:**
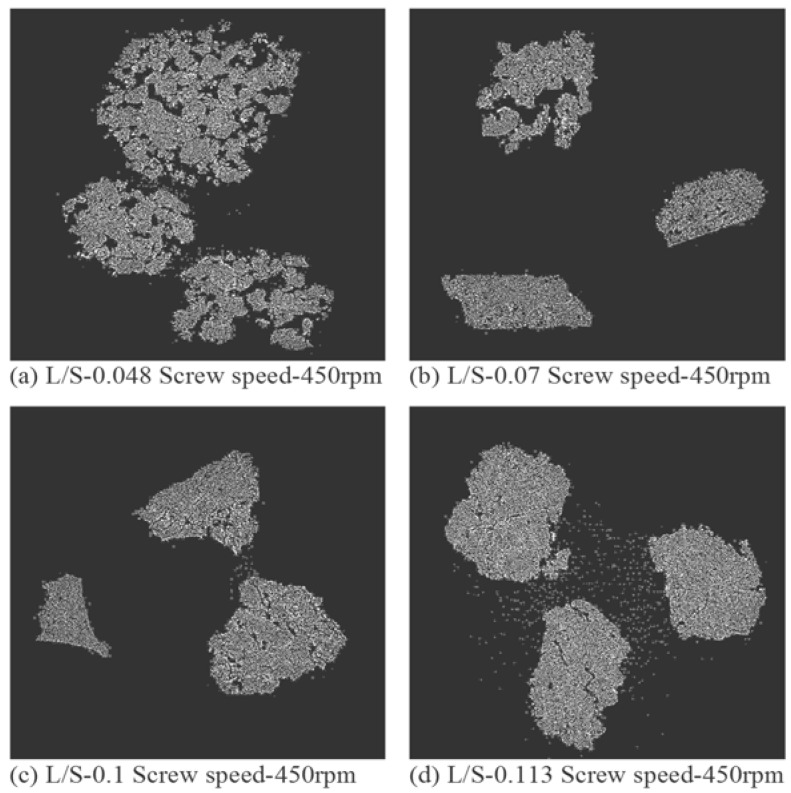
X-ray tomographic images of granules produced using Pearlitol 300DC at varying L/S (screw speed, 450 rpm).

**Figure 14:**
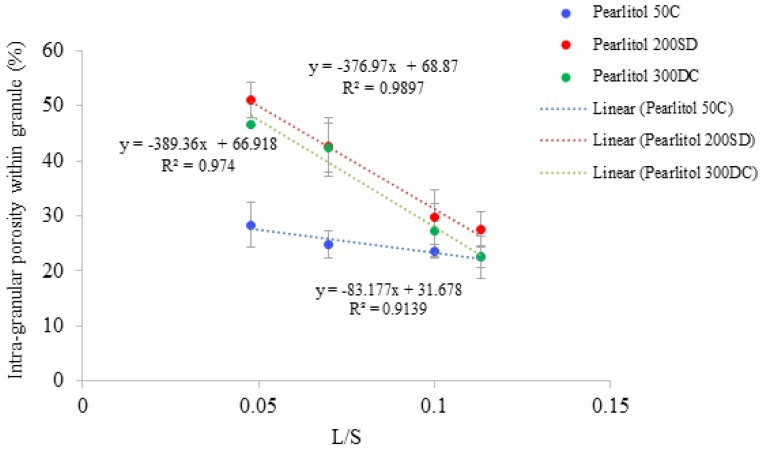
Porosity of granules for different grades of mannitol powder at varying L/S (screw speed, 450 rpm).

X-ray tomographic images of granules produced using different grades of mannitol are presented in Figure 11, Figure 12 and Figure 13. The respective intra-granular porosity within the granules calculated using ImageJ software is presented in Figure 14.

### 3.5. Tensile Strength of Tablets

Granules produced at L/S of 0.048 and 0.113 were sieved in size range of 212–1400 μm and into further subdivisions of 212–600 μm, 600–1000 μm, and 1000–1400 μm. This was done to study the effect of different granule size range on the tableting and tablet tensile strength.

#### 3.5.1. Pharmatose 200M

**Figure 15:**
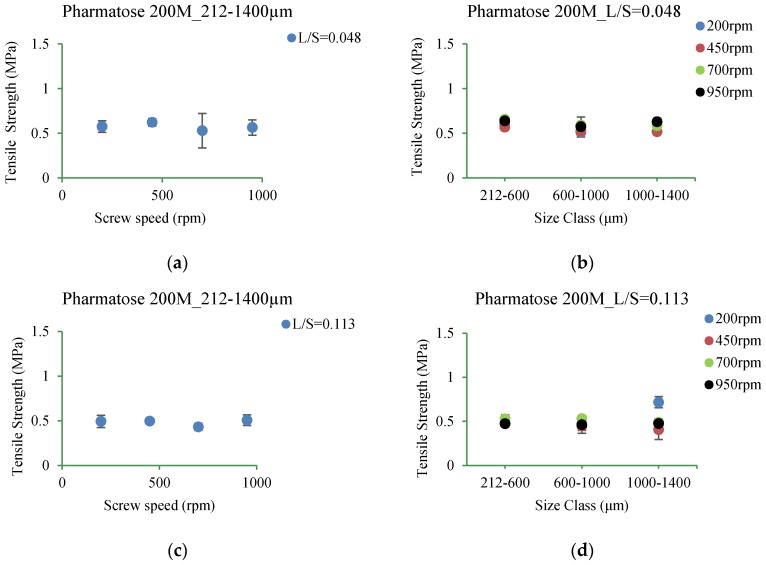
Tensile strength of tablet of granules at different size classes of Pharmatose 200M. (**a**) 212–1400 µm at L/S of 0.048; (**b**) 212–600 µm, 600–1000 µm, and 1000–1400 µm at L/S of 0.048; (**c**) 212–1400 µm at L/S of 0.113; (**d**) 212–600 µm, 600–1000 µm, and 1000–1400 µm at L/S of 0.113.

Figure 15a–d shows the tensile strength of tablets of Pharmatose 200M granules produced at L/S of 0.048 and 0.113 at different screw speeds (200 rpm, 450 rpm, 700 rpm, and 950 rpm) for different granule size ranges (complete range: 212–1400 μm; subdivisions: 212–600 μm, 600–1000 μm, 1000–1400 μm).

#### 3.5.2. SuperTab11SD

**Figure 16:**
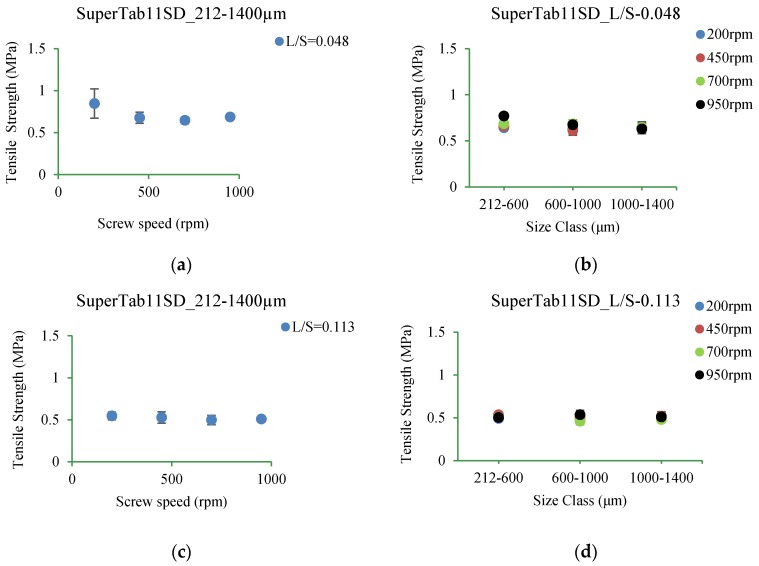
Tensile strength of tablet of granules at different size classes of SuperTab11SD. (**a**) 212–1400 µm at L/S of 0.048; (**b**) 212–600 µm, 600–1000 µm, and 1000–1400 µm at L/S of 0.048; (**c**) 212–1400 µm at L/S of 0.113; (**d**) 212–600 µm, 600–1000 µm, 1000–1400 µm at L/S of 0.113.

Figure 16a–d shows the tensile strength of tablets of SuperTab11SD granules produced at L/S of 0.048 and 0.113 (at varying screw speed) for different size classes. Like Pharmatose 200M, tablet strength was similar at all conditions.

#### 3.5.3. SuperTab21AN

**Figure 17:**
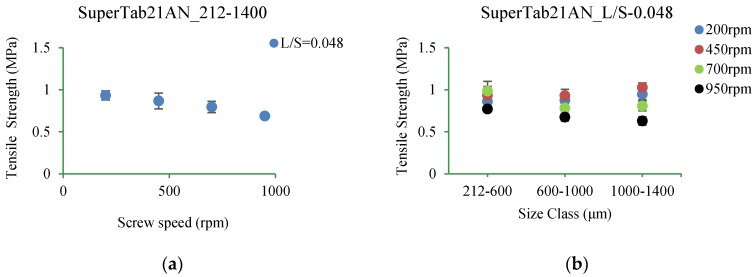

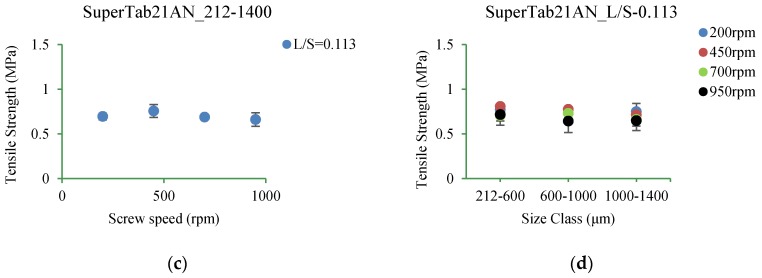
Tensile strength of tablet of granules at different size classes of SuperTab21AN. (**a**) 212–1400 µm at L/S of 0.048 (**b**) 212–600 µm, 600–1000 µm, 1000–1400 µm at L/S of 0.048 (**c**) 212–1400 µm at L/S of 0.113 (**d**) 212–600 µm, 600–1000 µm, 1000–1400 µm at L/S of 0.113.

Figure 17a–d shows the tensile strength of tablets of SuperTab21AN granules produced at L/S of 0.048 and 0.113 (at varying screw speed) for different size classes.

#### 3.5.4. Pearlitol 50C

**Figure 18:**
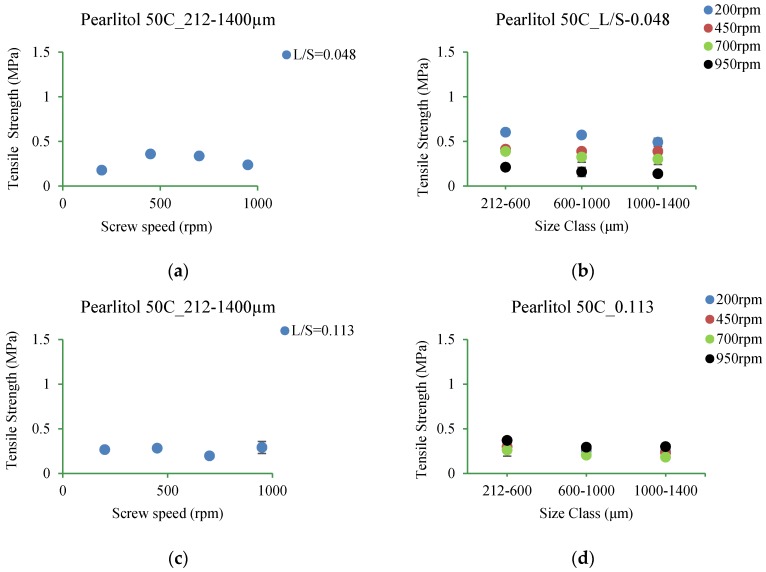
Tensile strength of tablet of granules at different size classes of Pearlitol 50C. (**a**) 212–1400 µm at L/S of 0.048; (**b**) 212–600 µm, 600–1000 µm, 1000–1400 µm at L/S of 0.048; (**c**) 212–1400 µm at L/S of 0.113; (**d**) 212–600 µm, 600–1000 µm, 1000–1400 µm at L/S of 0.113.

Figure 18a–d shows the tensile strength of tablet of Pearlitol 50C granules produced at varying L/S and screw speed for different size ranges.

#### 3.5.5. Pearlitol 200SD

**Figure 19:**
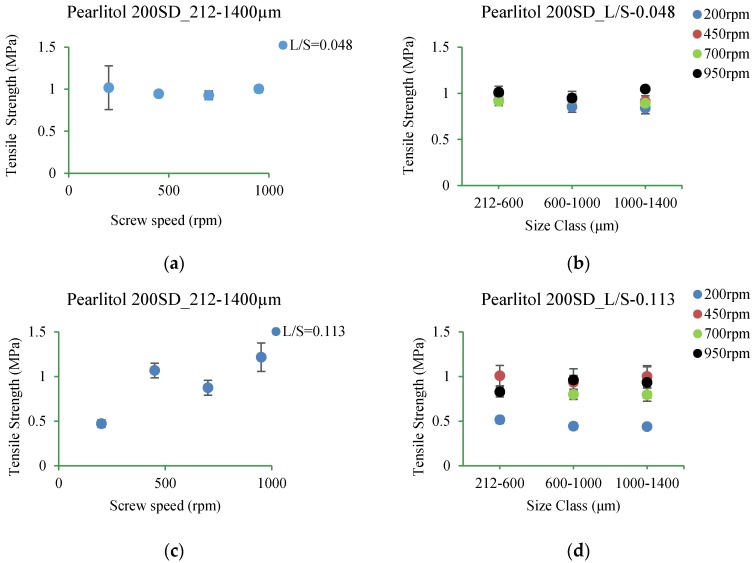
Tensile strength of tablet of granules at different size classes of Pearlitol 200SD. (**a**) 212–1400 µm at L/S of 0.048; (**b**) 212–600 µm, 600–1000 µm, 1000–1400 µm at L/S of 0.048; (**c**) 212–1400 µm at L/S of 0.113; (**d**) 212–600 µm, 600–1000 µm, 1000–1400 µm at L/S of 0.113.

Figure 19a–d shows the tensile strength of tablet of Pearlitol 200SD produced at varying L/S and screw speed for different size ranges.

#### 3.5.6. Pearlitol 300DC

**Figure 20:**
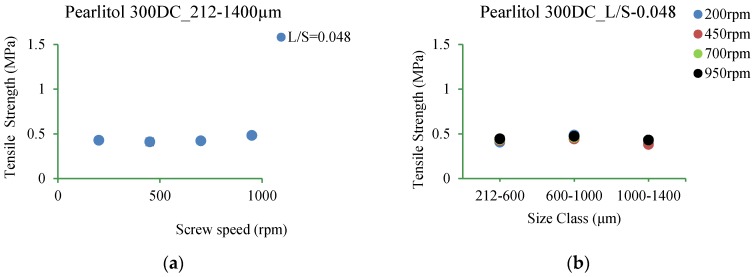

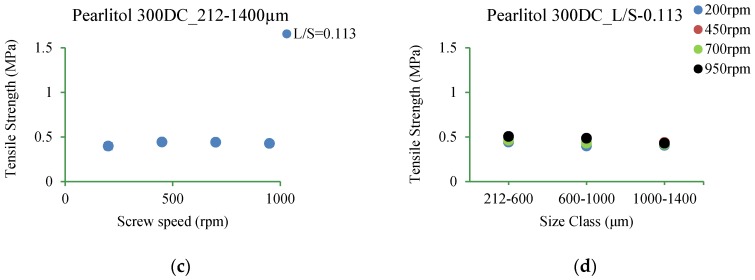
Tensile strength of tablet of granules at different size classes of Pearlitol 300DC. (**a**) 212–1400 µm at L/S of 0.048; (**b**) 212–600 µm, 600–1000 µm, 1000–1400 µm at L/S of 0.048; (**c**) 212–1400 µm at L/S of 0.113; (**d**) 212–600 µm, 600–1000 µm, 1000–1400 µm at L/S of 0.113.

Figure 20a–d shows tensile strength of tablets of Pearlitol 300DC produced at varying L/S and screw speed for different size ranges.

## 4. Discussion

### 4.1. Size of Granules

#### 4.1.1. Lactose

##### Effect of Varying L/S

From Figure 5a–d, it can be noticed that the granule size increased as the L/S increased for all three lactose powders. At the lowest L/S of 0.048, Pharmatose 200M had the smallest primary particle size (d_50_) amongst the three lactose powders, but it produced granules comparable to SuperTab21AN, which had larger primary particle size. This indicates that the smaller particles of Pharmatose 200M which promoted the dissolution of particle surface (more than anhydrous lactose [3]), potentially contributed to the formation of stronger liquid bridges between the particles, and thus to the granule growth [1,32,33]. As shown in Table 2, SuperTab21AN powder had larger primary particle size (d_50_-172.3 µm), and hence, limited dissolution of particle surface [3] and limited granule growth. At same L/S (i.e., 0.048) and screw speeds of 200 rpm and 450 rpm, SuperTab11SD which had larger and more compressible primary particles also produced similar sized granules. This may be because, at such low L/S, larger primary particles remained poorly wetted and were broken into smaller fragments owing to high compressibility, limiting the overall granule growth. No significant change in the granule size was observed at higher screw speeds of 700 rpm and 950 rpm (at the same L/S of 0.048). Although, some differences were observed amongst the three powders at various screw speeds, the overall maximum granule size was limited to ~650 µm due to low availability of liquid at L/S of 0.048. 

At L/S of 0.07 and screw speeds of 200 rpm and 450 rpm, SuperTab11SD produced relatively larger granules amongst the three powders. At screw speeds of 700 rpm and 950 rpm, SuperTab21AN had relatively larger median granule size amongst the three powders. This indicated that L/S of 0.07 and a screw speed of 700 rpm may be the parameter settings where SuperTab21AN started exhibiting granule growth. The presence of slightly more granulation liquid (compared to L/S of 0.048) with additional shear/compression force within TSG at higher screw speed resulted in the formation of stronger liquid bridges between SuperTab21AN particles, thus the increase in median granule size.

With further increase in the L/S to 0.1 and 0.113, the solubility of lactose powders increased further resulting in the formation of stronger and higher numbers of liquid bridges between primary particles, meaning formation of bigger and stronger granules. However, apart from the solubility, there are other factors such as compressibility which can also play a key role during wet granulation [1,33,34]. Pharmatose 200M is a crystalline lactose which is smaller in size and less compressible [1]. SuperTab11SD is a more compressible and spherical powder compared to the other two lactose powders. SuperTab21AN is also a crystalline lactose which is more compressible compared to Pharmatose 200M and less compressible compared to SuperTab11SD. At L/S of 0.1 and 0.113 (at all screw speeds except 200 rpm), SuperTab11SD produced the largest granules amongst all three powders. At such higher L/S, shearing action from the kneading elements promoted an even distribution of liquid into the SuperTab11SD powder. This liquid distribution helped to produce the plastic mass of powder, which promoted granules to coalesce and grow further rapidly at all screw speeds [18,35,36,37].

##### Effect of Varying Screw Speed

Figure 5a–d also shows that the increase in the screw speed had varying effects on granule size depending on the amount of liquid (L/S). In the case of Pharmatose 200M, the effect of increasing screw speed on the granule size was not prominent. This is in agreement with the observations by Dhenge, et al. [38] and Kumar, et al. [37] where varying screw speed (at lower throughput) resulted in a reduction in oversized granule fraction, and thereby increasing the granulation yield. According to Dhenge, et al. [38] and Kumar, et al. [37], varying screw speed changes the residence time and barrel fill level (surrogate for shear/frictional/compression forces), and thereby impacts the granule size. At lower screw speed, the barrel fill increases and the powder mass experiences a longer residence time. This, based on L/S and powder type, can increase or decrease the granule size. For Pharmatose 200M, all four L/S appeared to be sufficient to promote granule growth. Thus, at lower screw speed, granule growth occurred despite the presence of shear/frictional forces due to a high barrel fill level. As the screw speed increased, both barrel fill level and residence time decreased, but the axial mixing improved and granule–granule and granule–barrel wall impact increased, which resulted in granule growth as well as reduction in oversized granule fraction [37].

In the case of SuperTab11SD, at lower L/S of 0.048 and 0.07 (at all screw speeds), no significant difference was observed due to the porous structure of SuperTab11SD, where liquid was absorbed into the powder particles reducing the availability of liquid on the surface [39]. The absorbed interstitial liquid did not squeeze out sufficiently on the surface of granules due to low granule–granule and granule–barrel wall impact at lower screw speed. As mentioned earlier, at lower screw speed, barrel fill level and residence time also goes up [37] increasing the shear/frictional forces controlling the granule growth [38]. Increasing screw speeds at higher L/S of 0.1 and 0.113, granule size increased noticeably. This may be attributed to improved axial mixing and higher granule–granule and granule–barrel wall impact enhancing granule growth. Furthermore, these results can also be explained using a regime map described by Hapgood, et al. [12], where high liquid amount condition was described as “drop controlled regime”, while high shearing condition was indicated by “mechanical agitation controlled regime”.

In the case of SuperTab21AN, increasing screw speed at lower L/S of 0.048 did not show a significant effect on the granule growth, because liquid was not sufficient to form an adequate number of liquid bridges between the particles. When increasing screw speed from 200 rpm to 450 rpm at an L/S of 0.07, there was no significant change in granule size, but as the screw speed was increased further to 700 rpm and 950 rpm, the granule size increased noticeably. This is because at lower screw speeds 200 rpm and 450 rpm, the barrel fill level was high and residence time was longer [37,38]. This resulted in more attrition and breakage of formed granules which controlled the granule size. At higher screw speeds of 700 rpm to 950 rpm, the granule size increased because of high impact forces and improved axial mixing [37] which helped granules to squeeze out interstitial liquid on the surface promoting growth. Increasing screw speeds at higher L/S of 0.1 and 0.113 resulted in granule growth due to higher availability and better distribution of granulation liquid [18,35,36,37].

#### 4.1.2. Mannitol

##### Effect of Varying L/S

From Figure 6a–d it can be observed that increasing L/S had a powder dependent effect on the granule size. In the case of Pearlitol 50C and Pearlitol 200SD, granule size remained almost similar at L/S of 0.048–0.1 (at a screw speed of 200 rpm). As the L/S increased to 0.113, the granule size increased (Figure 6a) in both Pearlitol 50C and Pearlitol 200SD. Varying L/S at screw speeds of 450 rpm, 700 rpm, and 950 rpm resulted in no change in granule size for Pearlitol 50C and Pearlitol 200SD. In the case of Pearlitol 300DC, the granule size did not vary noticeably at L/S of 0.048 and 0.07, but increased at L/S of 0.1 and 0.113 (at all screw speeds).

Comparing the three powders, Pearlitol 50C, which has the smallest primary particle size (d_50_) amongst all mannitol powders, generally produced relatively larger granules in almost all cases, while Pearlitol 200SD produced small size granules. This was not the case with similar sized lactose powder (i.e., Pharmatose 200M (similar to Pearlitol 50C)) where granule size was either similar or smaller than the other two lactose powders at various conditions. Pearlitol 300DC, which has the largest primary particles amongst the three mannitol powders, produced granules comparable to Pearlitol 50C. This indicates that there are more dominant variable(s)/properties that controlled the granule size than the primary particle size of the powder. As discussed in the Section 3.1 and Section 3.2, the mannitol powders are significantly different in their size, shape, structure, and compressibility. One or more properties of the primary powder can dominate the granulation process outcome. For instance, Pearlitol 50C has the smallest size, elongated shape, and low compressibility. The small particle size of Pearlitol 50C potentially promoted the dissolution of particle surface contributing to the formation of stronger liquid bridges between the particles [1,19], and thus to the granule growth (similar to Pharmatose 200M). Pearlitol 200SD, on the other hand, had larger particles with porous structure and high compressibility. This means that the part of the liquid added may have been absorbed into the particle structure leaving an insufficient amount to promote the formation of sufficient numbers of liquid bridges between the particles, hence, very limited granule growth in the case of Pearlitol 200SD, even at moderately high L/S (0.07–0.113). This suggests that either the binding capacity of Pealrtiol 200SD is poor or it requires further increases in L/S (>0.113) to exhibit an increase in the granule size at increasing shear (i.e., at higher screw speeds). Additionally, the low compressibility factor (Figure 4) also supports the fact that Pearlitol 200SD particles are fragile [40]. Thus, the overall change in growth may be concealed by the volume reduction due to excellent compressibility and/or the breakage of poorly saturated particles. However, spray-dried lactose powder (SuperTab11SD), which has a similar compressibility factor to Pearlitol 200SD, produced larger granules at high L/S (0.1 and 0.113) at all four screw speeds. An attempt was made to understand this contrasting granule growth behavior of these two spray-dried powders (SuperTab11SD and Pearlitol 200SD) by determining differences between their drop penetration time, single particle strength, and dissolution rate (data not shown here). However, none of these additional measurements justified the difference in the granule growth behavior of SuperTab11SD and Pearlitol 200SD. This remains a topic for further investigation in the future.

Compared to Pearlitol 200SD, Pearlitol 300DC had the larger particle size; however, it is relatively low in compressibility (than the porous Pearlitol 200SD). It behaves like Pearlitol 50C at higher L/S. Pearlitol 300DC had large particles, but they are not porous, as in case of Pearlitol 200SD. This means that the liquid was not absorbed in the structure and was available for the inter-particle liquid bridge formation and thereby for granule growth. Additionally, there was less reduction in the volume due to relatively lower compressibility. Hence, it can be concluded that when there is significant difference in the structure and the compressibility, the granule growth is not just controlled by the primary particle size of powder in twin screw wet granulation. The effect on granule size can be a combination of one or more properties of a powder.

##### Effect of Varying Screw Speed

Figure 6a–d also shows the effect of screw speed on the median size of granules produced using the three mannitol powders. Increasing screw speed at all L/S had limited impact on the granule size.

### 4.2. Structure of Granules

X-ray tomography images of granules produced using different lactose powders at varying L/S (constant screw speed, 450 rpm) are shown in Figure 7, Figure 8 and Figure 9. The respective intra-granular porosity within the granules calculated using ImageJ software is presented in Figure 10. The XRT images and the respective calculated intra-granular porosity indicate that the granules generally densify linearly with increase in the L/S at a set screw speed in all three lactose powders. Comparing the XRT images of granules from the three powders, SuperTab11SD and SuperTab21AN granules appeared to be relatively more porous (due to higher inter-granular porosity) [41] due to larger primary particle size. However, they show localized densification (lower intra-granular porosity) within the granule structure owing to their porous primary particles and high compressibility.

Pearlitol 50C (Figure 11a–d) and Pearlitol 200SD (Figure 12a–d) showed less significant change in structure of larger granules at all L/S. However, few densified, elongated granules could be seen at L/S of 0.07–0.113 in the case of Pearlitol 200SD (Figure 12b–d). The intra-particle voids can also be seen in the XRT images. The XRT images support the granule size results for Pearlitol 200SD where the granule size did not change noticeably at increasing L/S and screw speed. The effect of L/S on granule structure was more obvious in the case of Pearlitol 300DC (Figure 13a–d), where granules became denser with increasing L/S. At L/S of 0.048 (Figure 13a), individual large-size particles of Pearlitol 300DC could still be identified in the granule structure. The intra-granular porosity within the granules (Figure 14) supported the observation from XRT images for Pearlitol 300DC. The intra-granular porosity within the granules (Figure 14) also supported the observation from XRT images for Pearlitol 50C where porosity did not vary significantly with increasing L/S. However, in the case of Pearlitol 200SD, the intra-granular porosity decreased with increasing L/S. This is due to the localized densification within the granules.

### 4.3. Tensile Strength of Tablets 

#### 4.3.1. Pharmatose 200M

It was found that the tensile strength of tablets was similar at both L/S of 0.048 and 0.113 (and at all screw speeds of 200 rpm, 450 rpm, 700 rpm, and 950 rpm) (Figure 15a–d). The granule size also had very limited impact on the tablet strength. It appears that compression force applied during tableting overcame the granule strength (at low or high L/S or screw speeds), thus a minimum effect on the tablet strength.

#### 4.3.2. SuperTab11SD 

The tensile strength of tablets of SuperTab11SD granules was similar at L/S of 0.048 and 0.113 (at varying screw speed) for different size classes (Figure 16a–d).

#### 4.3.3. SuperTab21AN

From Figure 17a–d it can be observed that the tensile strength of tablets of SuperTab21AN granules was similar to Pharmatose 200M and SuperTab11SD where it did not change (for broader granule size class of 212–1400 µm) with an increase in the L/S and screw speed (Figure 17a,c). The results agree with findings by Keleb, et al. [3], where tensile strength of tablets produced from milled lactose (Pharmatose 200M), spray-dried lactose (Pharmatose DCL 11), and anhydrous lactose (Pharmatose DCL 21) remained almost similar when wet granulated using batch high-shear granulator.

Subdividing the granules into narrower size classes showed a screw speed dependent effect on the tablet strength to some extent (Figure 17b). The tablet strength increased at lower screw speeds of 200 rpm and 450 rpm, while it decreased at higher screw speeds of 700 rpm and 950 rpm, with increasing granule size class from 212–600 µm to 600–1000 µm, and then to 1000–1400 µm. It may be interpreted that the granules in the size of 600–1000 µm and 1000–1400 µm, which are bigger (which are generally stronger according to Dhenge, et al. [42]), dictated the trend of decrease in the tablet strength with increasing screw speed in the broader granule size range (212–1400 µm). At higher L/S of 0.113, the effect of screw speed was concealed by the liquid amount available for the granulation.

#### 4.3.4. Pearlitol 50C

From Figure 18a–d, it can be seen that the tablets produced using broader granule size range (212–1400 µm) does not vary significantly upon increasing screw speed at both L/S (Figure 18a,c). But subdividing the granules from 212–1400 µm into 212–600 µm, 600–1000 µm and 1000–1400 µm showed effect of granule size on the tablet tensile strength at different screw speed (Figure 18b). Increasing the granule size range from 212–600 µm to 600–1000 µm and to 1000–1400 µm, decreased the tensile strength of the tablet [43,44]. The smaller granule size range (212–600 µm) produced stronger tablets [36,45]. This is because the small particles helped to make higher numbers of inter-particle bonds and pack better, making stronger tablets [45]. As granule size range increased to 600–1000 µm and to 1000–1400 µm, the number of bonds between the particles decreased resulting in weaker tablets. As the L/S increased to 0.113, the availability of sufficient liquid promoted the formation of stronger granules that masked the effect of screw speed.

#### 4.3.5. Pearlitol 200SD

In Pearlitol 200SD, varying screw speed at lower L/S of 0.048 showed no significant change in tablet tensile strength for broader (212–1400 µm) and subdivided granule size ranges (212–600 µm, 600–1000 µm and 1000–1400 µm) (Figure 19a,b). The outcome was similar at L/S of 0.113 except at condition at 200 rpm where tablet tensile strength was lower for both broader and subdivided granule size ranges (Figure 19c,d). This indicate that in presence of sufficient availability of granulation liquid (at L/S of 0.113), lower screw speed (meaning longer residence time and higher barrel fill) resulted in stronger granules, thus weaker tablets.

#### 4.3.6. Pearlitol 300DC

Figure 20a–d shows that the tensile strength of Pearlitol 300DC tablets was similar but very low at both lower and higher L/S at all screw speeds. 

It is clear that the tensile strength of tablets was low for all three mannitol powders at both lower and higher L/S at all screw speeds. It is likely that the differences in the granule strength or compressibility may have been concealed by the compression force used in the study [3].

## 5. Conclusions

Powders differ in their size and structure due to the way they are manufactured for their intended application (suitability for dry or wet granulation or direct compaction). In this study, the effects of the properties of powder on twin screw wet granulation behavior and properties of so-produced granules and tablets were studied. It was found that the granulation behavior of powder is controlled by size, structure, and compressibility of primary powder, which agrees with findings in previous research works [1,18,19,46]. The porous structure of a particle may enhance the compressibility in dry granulation or direct compaction, but in wet granulation it can limit the binding between the particles due to absorption of water in the structure of the particles. In general, increasing L/S resulted in an increase in the granule size and weaker tablets in all powders. The effect of screw speed on granule properties was dependent on the amount of liquid added and type of powder. Considering the granule size data, this work suggests that spray-dried lactose and anhydrous lactose can be used in twin screw wet granulation while operating at lower settings of L/S and screw speed. In case of mannitol, Pearlitol 200SD and 300DC are suitable for twin screw wet granulation at all L/S and screw speeds tested in this study. In general, processed powders such as spray-dried and granulated lactose and mannitol can also be used in formulation for wet granulation where flowability of active pharmaceutical ingredient (API) is poor. They may even be preferred over crystalline milled excipient powders (i.e., Pharmatose 200M and Pearlitol 50C, which have relatively poor flowability) while improving granule properties. In the future, the impact of twin wet granulation on the physicochemical characteristics of the granules and tablets of lactose and mannitol powders may be investigated to understand if they undergo form change due to wetting.

## Figures and Tables

**Table 1:** Experimental conditions and variables used in the study. L/S: liquid to solid ratios.

Experiment	Powder Type	Powder Feed Rate (kg/h)	Screw Speed (rpm)	L/S	Liquid Binder
Effect of powder type	Pharmatose 200M	2	200, 450, 700, 950	0.048, 0.07, 0.1, 0.113	Distilled water
	SuperTab11SD				
	SuperTab21AN				
	Pearlitol 50C				
	Pearlitol 200SD				
	Pearlitol 300DC				

**Table 2:** Primary particle size of powders used in the study.

Powder Grade	Particle Size (μm)		
	d_10_	d_50_	d_90_
Pharmatose 200M	9.3	42.1	110.0
SuperTab11SD	45.0	113.4	191.3
SuperTab21AN	27.5	172.3	330.0
Pearlitol 50C	10.1	33.9	114.9
Pearlitol 200SD	26.0	145.3	200.9
Pearlitol 300DC	58.3	249.9	385.9
